# Effects of Lecithin Supplementation in Feed of Different fat Levels on Serum Indexes and Liver Health of Laying Hens

**DOI:** 10.3389/fphys.2022.892585

**Published:** 2022-07-19

**Authors:** Gui-Li Hu, Juan Xiong, Yang Liu, Hong-Jun Yang, Ling-Ling Hu, Peng Chen, Xin Wang, Shuang Liao, Tuo Lv, Chun-Jie Liu, Peng Huang, Qian Lin

**Affiliations:** ^1^ Centree Bio-tech (Wuhan) Co., LTD, Wuhan, China; ^2^ Institute of Bast Fiber Crops, Chinese Academy of Agricultural Sciences, Changsha, China

**Keywords:** soy lecithin, biochemical indexes, antioxidant index, laying performance, liver health

## Abstract

The aim of this experiment was to investigate the effect of soy lecithin on serum-related indicators and liver health in laying hens under the influence of high-fat diets. 180 peak laying hens at 40 weeks of age were randomly assigned to one of the four diets using a 2 × 2 factorial and fed for 5 weeks. The results showed that compared to the low-fat group, the high-fat group had lower egg production (*p* < 0.05) and higher average daily feed intake and feed-to-egg ratio (*p* < 0.05). At the 21^st^ day, the serum levels of triglyceride (TC) and superoxide dismutase (SOD) were higher (*p* < 0.05), high-density lipoproteins cholesterol (HDL-C) levels were lower (*p* < 0.01), catalase (CAT) activity was lower (*p* < 0.05), TC and malondialdehyde (MDA) levels in liver were higher (*p* < 0.01) and SOD activity in liver was lower (*p* < 0.05) in layers supplemented with soy lecithin. CAT activity in serum was increased (*p* < 0.01) and total antioxidant capacity (T-AOC) activity in the liver was decreased (*p* < 0.05) after increasing the dietary fat concentration. The addition of soy lecithin and the increase in dietary fat concentration had a highly significant interaction on serum CAT activity and liver TC content in layers (*p* < 0.01). At the 35^th^ day, the serum alanine aminotransferase (ALT) activity was higher (*p* < 0.01), serum glutathione peroxidase (GSH-Px) and CAT activity were higher (*p* < 0.05), and serum triglyceride (TG) content and total T-AOC capacity activity were lower (*p* < 0.05) in layers supplemented with soy lecithin. Increasing dietary fat concentration decreased alanine aminotransferase (ALT), aspartate aminotransferase (AST) and GSH-Px activity in serum (*p* < 0.05). However, it increased TG and MDA content in liver (*p* < 0.05), and highly decreased SOD content in liver (*p* < 0.01) in layers. The addition of soy lecithin and increasing dietary fat concentration had a highly significant reciprocal effect on serum ALT viability and CAT viability (*p* < 0.01) and liver TG and MDA content and SOD viability (*p* < 0.05) in layers. In conclusion, feeding high-fat diets will adversely affect the laying performance of laying hens, while long-term addition of lecithin can improve the blood lipids and liver lipids of laying hens, enhance the antioxidant capacity of the liver, and maintain liver health.

## Introduction

With the rapid development of the animal husbandry industry, the breeding environment became more and more complex, which potentially causes various diseases. As the most important detoxification organ in the body, the liver plays an important role. It has been observed that in the long-term breeding process of laying hens, the abdomens of some hens were enlarged and soft, and their egg production declined. In hens that died unexpectedly, postpartum revealed hemorrhages and liver damage, all of which were caused by fatty liver disease (Liu, 2017). The liver health of livestock and poultry is positively related to their growth. Liver damage often occurs during the breeding process. Therefore, it requires more attention to repair the liver damage.

Soy lecithin is mainly a mixture of various phospholipids, including phosphatidylcholine, phosphatidylinositol, phosphatidylethanolamine, etc., (Scholfield, 1981). The active ingredients contained in soy lecithin play an important role in maintaining the integrity, fluidity, and functions of cell membranes (Pagheh, et al., 2018). It was reported that under carbon tetrachloride modeling, phospholipids could slow down the increase in liver weight, promote the regeneration of hepatocytes, and gradually restore the fascicular and lobular structures of the liver (Drozdov, 2014). There were also reports suggesting that adding lecithin to the diet could improve the pathological changes in the liver of laying hens, as well as the abnormal expression of apoA I and apoB100 genes in the liver (Yalu et al., 2017). It has also been found that supplementation of 0.5–2.0% soybean lecithin (SL) improved the production performance of laying hens and total phospholipid contents in whole eggs and egg yolks were also increased at 1, 2, and 4 and 2% SL supplementation than the control group, respectively (Sun et al., 2010). Further, Lu et al. (2018) also found that supplementation of 0.1 and 0.2% lecithin in high-fat diets improved the laying hens’ egg production rate and reduced liver index, blood fat content, and occurrence of fatty liver syndrome. However, limited research is available on the effect of lecithin on the liver health of laying hens. Therefore, this study examined the effects of dietary fat concentration and lecithin on serum markers and liver health in laying hens.

## Materials and Methods

### Test Material

The lecithin (acetone insoluble content ≥ 90%) used in this study was provided by Centree Bio-tech (Wuhan) Co., Ltd. China.

### Animals and Experimental Details

All the experimental procedures were approved by the Animal Care Committee of the Institute of Bast Fiber Crops, Chinese Academic of Agricultural Sciences, Changsha, China. A total of 180 Lohmann Commercial laying hens (40-week-old) were randomly divided into four groups, with five replicates of 9 birds each. Experimental laying hens were pre-fed for 30 days and rearranged after confirmation of no clinical abnormality. A 2 × 2 factorial was randomly assigned to 4 groups with the basal diet was a corn-soybean meal diet, the dietary fat rate of 1 and 6% and dietary supplemented lecithin concentration of 0 and 1 kg/t as the main treatment factors. The normal dietary protein level was 16.5% and the high-fat diet protein level was 13.5%. Other nutrient levels met the recommendations of the National Research Council (NRC) for laying hens (1994) and “NY/T 33–2004 Chicken Breeding Standards” as shown in Table 1. The hens were raised in ladder cages with one bird in each cage. After 4 weeks of the adaptation period, the main experiment started and lasted for 7 weeks. The egg production, body weight, and feed intake of each laying hen were measured on the first day of the experiment. No statistical differences in the production performance were found among treatments. Free water and feed were provided for all hens. The average temperature was 25 ± 2°C in the laying hens’ house during the experimental period. The light time was according to the standard light procedure of commercial laying hens, which was 16 h of light per day, until the end of the experiment.

**TABLE 1:** Composition and nutrient levels of experimental diets (air-dried basis) %.

Items	Diets	
	Basal diet	High-fat diet
Ingredients		
Corn	58.08	57.52
Soybean meal	28.37	20.16
Rice husk	0.00	0.51
Oil	1.08	6.10
Wheat bran	1.00	4.20
Limestone	8.47	8.51
CaHPO_4_·2H_2_O	0.00	0.00
98.0% *L*- Lys	0.00	0.00
98.5% *DL*- Met	0.00	0.00
NaCl	0.00	0.00
3% Premix1)	3.00	3.00
Total	100.00	100.00
Nutrient levels2)		
ME/(Mcal/kg)	2.75	3.00
CP	16.50	13.50
CF	3.05	3.05
Ca	3.50	3.50
Total *p*	0.54	0.53
Available *p*	0.32	0.32
NaCl	0.31	0.31

1) The premix provided the following (per kilogram of complete diet) micronutrients: VA, 6 000 IU, VD3 2 500 IU, VE, 25 mg, VK3 2.25 mg, VB1 1.8 mg, VB2 7 mg, VB6 4 mg, VB12 0.2 mg, D-pantothenic acid 12 mg, nicotinic acid 35 mg, biotin 0.14 mg, folic acid 0.8 mg, Cu (as copper sulfate) 11 mg, Zn (as zinc sulfate) 70 mg, Fe (as ferrous sulfate) 60 mg, Mn (as manganese sulfate) 115 mg, Se (as sodium selenite) 0.30 mg, I (as potassium iodide) 0.4 mg.

2) Nutrient levels are calculated values.

### Egg Production Performance

The laying hens of each treatment were fed with the corresponding diet according to the experimental design. The feed intake was recorded. The egg production of chickens was recorded and each egg was weighed. The deaths were also recorded every day.

### Determination of Serum Biochemical Indexes and Antioxidant Indexes

On the morning of the 21^st^ and the 35^th^ day, 5 test chickens were randomly selected from each treatment (1 bird per replicate), and about 8 ml of blood were collected from the wing vein after weighting with 10 ml centrifuge tubes and placed in a tilted position to allow the blood to coagulate naturally. After 0.5 h of hemagglutination, the blood samples were centrifuged at 3000 r/min for 15 min to separate the serum. The upper serums were collected and stored at −40°C. Serum biochemical indices, including triglyceride (TG), cholesterol (TC), aspartate aminotransferase (AST), alanine aminotransferase (ALT), low-density lipoprotein cholesterol (LDL-C), and high-density lipoprotein cholesterol (HDL-C) were measured by using commercial kits following the instructions assay kits (BS-200, Shenzhen Mairui Medical International Co., Ltd., China). Antioxidant indexes, including total antioxidative capacity (T-AOC), catalase (CAT), superoxide dismutase (SOD), glutathione peroxidase (GSH-Px), and malonaldehyde (MDA) were examined using assay kits (H249, Nanjing Jiancheng Bioengineering Institute, China) with an automated fluorescence instrument (MultiskanSkyHigh, Thermo Fisher Scientific, Waltham, MA, United States) following the manufacturers’ instructions.

### Detection of Liver Indexes

The liver indexes were calculated after peeling and weighing the livers of the slaughtered chickens. Three samples of suitable size were collected from each liver sample. They were cut in the middle of the large liver lobe, wrapped in tin foil, and stored at −80°C in refrigerator. Afterwards, 0.3 g of the liver sample was taken and homogenized with cold physiological saline using an Ultra-Turrax homogenizer from Tekmar (Cincinnati, United States), followed by centrifugation at 4000 × g for 15 min at 4°C for indexes analysis. Antioxidant indexes in the liver, including T-AOC, CAT, SOD, GSH-Px, and MDA were examined using assay kits (H249, Nanjing Jiancheng Bioengineering Institute, China) with an automated fluorescence instrument (MultiskanSkyHigh, Thermo Fisher Scientific, Waltham, MA, United States) following the manufacturers’ instructions.

### Data Processing and Statistical Analysis

After the experimental data was preliminarily processed with Excel 2007 software, two-way ANOVA was performed with SPSS 19.0 statistical software. *p* < 0.05 and 0.01 suggested significant differences and extremely significant differences respectively. The test results were expressed as the mean of each group. The dataset standard error (SEM) results were also presented.

## Results

### Laying Performance

It could be seen from Table 2 that on the 35^th^ day, the egg production rate, average egg weight, average daily feed intake, and the egg mass/feed consumption ratio between the layers treated with or without lecithin were not affected (*p* > 0.05). Compared with the low-fat group, the laying rate of the hens in the high-fat group was decreased (*p* < 0.05). The average daily feed intake and the egg mass/feed consumption ratio were increased (*p* < 0.05). The average egg weight had no change (*p* > 0.05). The interactions of lecithin and fat on egg production rate, average egg weight, average daily feed intake, and the egg mass/feed consumption ratio were not affected (*p* > 0.05).

**TABLE 2:** Effect of adding lecithin on egg laying performance of laying hens on 35 days.

Items		1% Fat		6% Fat			Lecithin		Fat		*p*- value		
	Lecithin	0 kg/t	1 kg/t	0 kg/t	1 kg/t	SEM	0 kg/t	1 kg/t	1%	6%	Fat	Lecithin	Interaction
Egg production rate (%)		96.63	96.41	91.60	88.92	1.22	94.12	92.67	96.52	90.26	0.045	0.607	0.663
Average egg weight (g)		60.96	59.22	59.68	60.88	0.40	60.32	60.05	60.09	60.28	0.846	0.790	0.167
Average daily feed intake (g)		116.08	116.83	125.49	125.57	1.60	120.79	121.20	116.46	125.53	0.010	0.887	0.910
The egg mass/feed consumption ratio		1.90	1.97	2.11	2.06	0.02	2.01	2.02	1.94	2.08	0.001	0.704	0.108

In the same row, values with no letter or the same letter superscripts mean no significant difference (*p* > 0.05), with different small letter superscripts mean significant difference (*p* < 0.05), and with different capital letter superscripts mean extremely significant difference (*p* < 0.01). The same as below.

### Serum Biochemical Indicators

It could be seen from Table 3 that on the 21^st^ day, the content of TC in the serum of the group treated with lecithin was increased (*p* < 0.05) compared to the treatment group without lecithin, whereas the content of HDL-C was decreased (*p* < 0.01). The content of HDL-C showed an upward trend (*p* = 0.081), while the activities of ALT, AST, and TG in serum had no differences (*p* > 0.05). Compared with the low-fat group, the serum ALT and AST activities, TG, TC, HDL-C, and LDL-C contents of laying hens in the high-fat group had no difference (*p* > 0.05). The interactions of lecithin and fat on ALT, AST activities, and TG, TC, HDL-C, and LDL-C content in laying hens’ serum were not affected (*p* > 0.05).

**TABLE 3:** Effects of adding lecithin on serum biochemical indexes of laying hens.

Items			1% Fat		6% Fat			Lecithin		Fat		*p*- value		
		Lecithin	0 kg/t	1 kg/t	0 kg/t	1 kg/t	SEM	0 kg/t	1 kg/t	1%	6%	Fat	Lecithin	Interaction
	ALT (U/L)		1.89	2.10	2.15	2.31	0.09	2.02	2.2	2	2.23	0.228	0.335	0.897
	AST (U/L)		18.24	19.81	16.89	18.14	0.53	17.56	18.98	19.03	17.51	0.160	0.186	0.879
21d	TC (mmol/L)		23.99	28.15	22.22	27.79	1.45	23.1	27.97	26.07	25	0.718	0.112	0.811
	TG (mmol/L)		3.25	3.84	2.67	3.55	0.18	2.96	3.69	3.54	3.11	0.206	0.038	0.655
	HDL-C (mmol/L)		0.41	0.30	0.36	0.31	0.01	0.39	0.31	0.36	0.34	0.443	0.002	0.143
	LDL-C (mmol/L)		1.35	1.69	1.47	1.8	0.09	1.41	1.74	1.52	1.63	0.535	0.081	0.971
													
	ALT (U/L)		2.22^A^	2.20^A^	1.37^B^	2.25^A^	0.11	1.79	2.22	2.21	1.81	0.010	0.007	0.005
	AST (U/L)		18.17	20.95	16.41	16.79	0.62	17.29	18.87	19.56	16.60	0.010	0.138	0.252
35d	TC (mmol/L)		31.74	26.97	35.43	29.33	1.61	33.59	28.15	29.35	32.38	0.348	0.102	0.835
	TG (mmol/L)		4.18	3.30	4.01	3.54	0.17	4.10	3.42	3.74	3.77	0.923	0.048	0.538
	HDL-C (mmol/L)		0.32	0.33	0.25	0.36	0.02	0.28	0.34	0.32	0.30	0.741	0.222	0.318
	LDL-C (mmol/L)		1.56	1.48	1.57	1.38	0.06	1.56	1.43	1.52	1.48	0.728	0.263	0.655

ALT, alanine aminotransferase; AST, aspartate aminotransferase; TC, triglyceride; TG, Total cholesterol; HDL-C, high density liptein cholesterol and LDL-C, low-density lipoprotein cholesterol.

On the 35^th^ day, compared to those without the lecithin supplementation treatment group, the serum ALT activity was highly increased (*p* < 0.01). The TC content was decreased (*p* < 0.05) in the lecithin supplemented treatment group. But the serum AST activity, contents of TG, HDL-C, and LDL-C had no difference (*p* > 0.05). Compared with the low-fat group, the serum AST and ALT activities of the laying hens in the high-fat group were decreased (*p* < 0.01), whereas the serum AST activity, TC, TG, HDL-C, and LDL-C contents had no difference (*p* > 0.05). There was an interaction between lecithin and ALT activity in the serum of laying hens (*p* < 0.05). But the interactions on the contents of AST, TG, TC, HDL-C, and LDL-C in serum were not affected (*p* > 0.05).

### Serum Antioxidant Indicators

It could be seen from Table 4 that on the 21^st^ day, compared with the treatment group without lecithin supplementation, the CAT activity in the serum was decreased (*p* < 0.01), whereas the SOD, GSH-Px, T-AOC in the serum were significantly reduced (*p* < 0.01) in the lecithin supplemented treatment group. But the MDA content had no difference (*p* > 0.05). Compared with the low-fat group, the activities of SOD and CAT in the serum were increased (*p* < 0.05), and the activity of GSH-Px was decreased (*p* = 0.080) in the laying hens in the high-fat group. There was an interaction between lecithin and fat on the activity of CAT in the serum of laying hens (*p* < 0.01). But the interactions on the activities of SOD, GSH-Px, and T-AOC and the content of MDA in serum were not affected (*p* > 0.05).

**TABLE 4:** Effects of adding lecithin on serum Antioxidant Index of Laying Hens.

Items			1% Fat		6% Fat			Lecithin		Fat		*p*- value		
		Lecithin	0 kg/t	1 kg/t	0 kg/t	1 kg/t	SEM	0 kg/t	1 kg/t	1%	6%	Fat	Lecithin	Interaction
21d	T-SOD (U/mgprot)		124.42	130.75	124.02	149.19	4.12	124.22	139.97	127.59	136.61	0.239	0.049	0.220
	MDA (nmol/mgprot)		7.49	8.94	7.71	8.50	0.55	7.60	8.72	8.21	8.11	0.927	0.348	0.777
	GSH-PX (U/mgprot)		462.44	739.44	572.99	562.70	39.9	517.71	651.07	600.94	567.84	0.649	0.080	0.061
	CAT (U/mgprot)		2.40B	1.31C	2.93AB	3.08A	0.18	2.67	2.20	1.86	3.01	< 0.001	0.035	0.008
	T-AOC (mmol/gprot)		1.05	1.26	1.31	1.17	0.07	1.18	1.22	1.16	1.24	0.550	0.779	0.232
													
35d	T-SOD (U/mgprot)		135.73	131.52	131.06	138.36	4.04	133.39	134.94	133.63	134.71	0.902	0.861	0.517
	MDA (nmol/mgprot)		10.97	7.47	11.10	10.90	0.60	11.04	9.18	9.22	11.00	0.109	0.096	0.134
	GSH-PX (U/mgprot)		794.06	1004.68	711.07	780.61	37.10	752.56	892.65	899.37	745.84	0.020	0.031	0.252
	CAT (U/mgprot)		1.18B	3.22A	2.46A	2.16A	0.24	1.82	2.69	2.20	2.31	0.765	0.032	0.006
	T-AOC (mmol/gprot)		1.55	1.21	1.49	1.40	0.06	1.52	1.30	1.38	1.45	0.528	0.044	0.237

T-SOD, total superoxide dismutase; MDA, malondialdehyde; GSH-PX, glutathione peroxidase; CAT, catalase and T-AOC, total antioxidant capacity.

On the 35^th^ day, compared to without lecithin supplemented treatment group, the serum GSH-Px activity in the lecithin supplemented treatment group was increased (*p* < 0.01), whereas the SOD, CAT, T-AOC activity, and MDA content in the serum did not show differences (*p* > 0.05). Compared with the low-fat group, the serum GSH-Px and CAT activities of the laying hens were decreased (*p* < 0.05), the T-AOC activity was increased (*p* < 0.05), and the serum MDA content had a tendency to increase (*p* = 0.096) in the high-fat group. There was no change in SOD activity in serum (*p* > 0.05). Besides, there was an interaction between lecithin and fat in the activity of CAT in the serum of laying hens (*p* < 0.01), but the interactions in the activities of SOD, T-AOC, GSH-Px, and MDA content in serum were not obvious (*p* > 0.05).

### Liver Index and Liver Triglyceride Content

It could be seen from Table 5 that on the 21^st^ and the 35^th^ day, the liver index of the laying hens of the four groups was not affected (*p* > 0.05). The interactions between fat content and lecithin on the liver index of laying hens did not reach a level (*p* > 0.05).

**TABLE 5:** Effects of adding lecithin on layer Liver Index and TG content of liver in laying hens.

Items			1% Fat		6% Fat			Lecithin		Fat		*p*- value		
		Lecithin	0 kg/t	1 kg/t	0 kg/t	1 kg/t	SEM	0 kg/t	1 kg/t	1%	6%	Fat	Lecithin	Interaction
21d	Liver index (%)		2.250	2.380	2.270	2.040	0.05	2.260	2.210	2.320	2.150	0.121	0.634	0.092
	TG (mmol/gprot)		0.5^B^	0.88^A^	0.65^B^	0.63^B^	0.04	0.58	0.76	0.69	0.64	0.414	0.008	0.003
														
35d	Liver index (%)		2.530	2.460	2.190	2.390	0.08	2.360	2.430	2.500	2.290	0.203	0.684	0.407
	TG (mmol/gprot)		0.93^B^	0.79^B^	0.87^B^	1.18^A^	0.05	0.90	0.98	0.86	1.02	0.040	0.260	0.010

It could be seen from Table 5 that on the 21^st^ day, compared with the treatment group without lecithin supplementation, the TG content in the liver of the lecithin supplemented treatment group was increased (*p* < 0.01). Compared with the low-fat group, the high-fat group had no difference on the liver TG content of laying hens (*p* > 0.05). However, lecithin and fat had an interaction on the liver TG content of laying hens (*p* < 0.01). On the 35^th^ day, the hepatic TG content of the lecithin supplemented treatment group and without lecithin supplemented group had no difference (*p* > 0.05). Compared with the low-fat group, the TG content in the liver of the laying hens in the high-fat group was increased (*p* < 0.01). Besides, there was an interaction between lecithin and fat on the TG content in the liver of the laying hens (*p* < 0.05).

### Liver Antioxidant Indicators

It could be seen from Table 6 that on the 21^st^ day, compared with the treatment group without lecithin supplementation, the SOD activity was decreased, and the MDA content was increased (*p* < 0.05) in the liver of the lecithin-added treatment group. There was no change in the activity of T-AOC (*p* > 0.05). Compared with the low-fat group, the liver T-AOC activity decreased (*p* < 0.05), and the MDA content increased (*p* < 0.01) of the laying hens in the high-fat group. There were no interactions between lecithin and fat on the measured antioxidant indexes in the liver of laying hens (*p* > 0.05).

**TABLE 6:** Effects of adding lecithin on antioxidant index of liver of laying hens.

Items			1% Fat		6% Fat			Lecithin		Fat		*p*- value		
		Lecithin	0 kg/t	1 kg/t	0 kg/t	1 kg/t	SEM	0 kg/t	1 kg/t	1%	6%	Fat	Lecithin	Interaction
21d	SOD (U/mgprot)		1089.98	961.08	1026.33	985.63	14.98	1058.16	973.36	1025.53	1005.98	0.381	0.001	0.059
	MDA (nmol/mgprot)		0.30	0.42	0.37	0.44	0.02	0.34	0.43	0.36	0.41	0.099	0.004	0.464
	GSH-PX (U/mgprot)		15.62	15.34	15.82	18.26	0.58	15.72	16.8	15.48	17.04	0.182	0.348	0.241
	CAT (U/mgprot)		5.77	7.46	8.24	9.36	0.65	7.01	8.41	6.62	8.8	0.103	0.282	0.823
	T-AOC (mmol/gprot)		0.23	0.21	0.20	0.20	0.01	0.22	0.20	0.22	0.20	0.045	0.141	0.419
													
35d	SOD (U/mgprot)		895.97^b^	995.91^a^	846.23^b^	819.98^b^	19.08	871.10	907.95	945.94	833.11	< 0.001	0.152	0.020
	MDA (nmol/mgprot)		0.57^b^	0.48^c^	0.68^a^	0.72^a^	0.03	0.62	0.60	0.52	0.70	< 0.001	0.407	0.030
	GSH-PX (U/mgprot)		17.27	20.91	18.52	20.55	0.83	17.89	20.73	19.09	19.53	0.793	0.105	0.631
	CAT (U/mgprot)		8.21	7.39	8.76	6.51	0.49	8.49	6.95	7.80	7.63	0.862	0.133	0.470
	T-AOC (mmol/gprot)		0.18	0.20	0.19	0.22	.010	0.18	0.21	0.19	0.20	0.314	0.084	0.825

On the 35^th^ day, compared to the without lecithin supplementation group, the hepatic T-AOC activity in the lecithin supplemented treatment group had a tendency to increase (*p* = 0.084), while the SOD, GSH-Px, CAT activity and MDA content in the liver were not affected (*p* > 0.05). Compared with the low-fat group, the SOD activity was significantly decreased, and the MDA content was increased (*p* < 0.01) in the liver of the laying hens in the high-fat group, while the activities of GSH-Px, CAT, and T-AOC in the liver had no differences (*p* > 0.05). There were interactions between lecithin and fat on the hepatic SOD activity and MDA content in laying hens (*p* < 0.05).

## Discussion

The purpose of fat addition in the diet for laying hens was mainly to reduce the pulverization of the feed to improve feed utilization. But high levels of dietary fat increased egg masses and laying hen body weights (Grobas et al., 2001). In the present study, it has been observed that with the increase in dietary fat concentration, the egg production decreased, whereas egg mass/feed consumption ratio of laying hens increased significantly. This was consistent with the previous report by Weiss and Fisher (1957) showing that a high level of animal fat resulted in decreased egg production and increased laying hen body weight. Zhang et al. (2008) also found that increased dietary fat concentration reduced the laying rate of laying hens. The decrease in egg production might be due to fat deposition in the abdomen because of a high energy fat diet, which increased the weight and created an excessive burden on the production performance of the layer (Li et al., 2009). In the current study, it was found that the lecithin supplementation in diet had no significant effect on the laying performance of laying hens, and the interaction between dietary lecithin and fat on the laying performance was not significant. A previous study by Attia et al*.* (2009) also revealed no significant changes in laying performance with lecithin supplementation. Further, our study also revealed no significant effects of dietary lecithin on the liver index of laying hens on the 21^st^ and 35^th^ days. A study by Mandalawi et al. (2015) also revealed that no significant effect on liver size in laying hens with lecithin supplementation in diets between 23 and 55 weeks of age.

In this study, it was found that on the 35^th^ day, the serum ALT activity was higher in the high-fat group, and the high-fat + lecithin group than in the diet supplemented with lecithin. The high levels of ALT activity in the high-fat group might be happened due to feeding a high-fat diet to laying hens for a long time which can result in an increased burden on the liver and can cause liver damage, especially in the late stage, because of the long growth cycle and high egg production pressure in the layer farming industry. Fatty liver hemorrhage syndrome is a common disease of laying hens, with an incidence rate of 5–30% (Guo et al., 2021). When the liver is damaged, the permeability of the liver cell membrane increases, and a variety of enzymes in the liver cells are released into the blood, such as ALT and AST. Therefore, the activities of these enzymes in the blood would increase (Cray et al., 2008). In poultry, the liver is involved in fat metabolism (Zaefarian et al., 2019). So, Increasing the dietary fat can increase the metabolic burden on the liver resulting in liver damage (Charradi et al., 2013). A previous study also showed that a high-fat diet could induce fatty liver, and the activities of ALT and AST in serum were significantly increased compared with the normal diet group (Zhang et al., 2008). Lecithin is the major component of cell membranes and can play a key role in cell repair and liver health. The results of the current study showed that on the 21^st^ day, dietary lecithin increased the content of TC, and LDL-C, whereas decreased the content of HDL-C in serum. As a surfactant, soybean lecithin could emulsify fat and might affect the absorption of fatty acids in the small intestine (Jenkins et al., 1989). It was also reported that soybean lecithin could promote the secretion of endogenous bile acids and improve the utilization of fat (Liu et al., 2020), thereby increasing the fat content in the blood. But on the 35^th^ day, the TC content in serum was significantly reduced by dietary lecithin. Soybean lecithin contains phosphatidylcholine, which is an important component of lipoprotein and plays an essential role in the process of lipid metabolism. Exogenous soybean lecithin could accelerate the decomposition of liver fat in laying hens resulting in a decrease in TC (Shen et al., 2021). Siyal et al. (2017) and Li et al. (2015) found that adding medium and high doses of lecithin to the feed could reduce the content of TC in the serum of broilers. The main active ingredient in lecithin was phosphatidylcholine. Lecithin metabolism releases phosphatidylcholine into the blood, which acts as a substrate for the conversion of cholesterol into cholesterol ester in the body and is associated with the stability of apolipoprotein. Moreover, lecithin has good hydrophilic, lipophilic, and emulsifying properties, so that it can convert cholesterol from large particles to small particles which can be easily absorbed by the tissues via the blood vessel wall, which ultimately decreases the blood lipids levels (Li et al., 2015). It was found in the current study that when laying hens were fed with the high-fat diet for 35 days, the TG content in the liver increased significantly, indicating that the high-fat diet increased the deposition of fat in the liver and increase the incidence of fatty liver. The addition of lecithin decreased the hepatic TG content compared with the high-fat diet group, which showed that lecithin could reduce the deposition of TG in the liver.

Laying hens are usually reared for a longer time duration to get maximum egg production. This can results in an accelerated oxidative rate in laying hens and oxidative damage is severe in the peak laying period. Continuous high production and vigorous metabolism increase the content of active oxygen free radicals in the body. This results in decreased activities of the antioxidant enzymes in laying hens, causing the excessive accumulation of the oxygen free radicals in the body. Excessive deposition of fat in the liver can trigger lipid peroxidation, which damages the liver (Zhao et al., 1995). A study in mice by Tm et al. (2020) showed that long-term feeding of a high-fat diet resulted in an increase in MDA content in the liver and a decrease in the activities of T-AOC, T-SOD, and GSH-Px in the liver. In this study, it was found that the content of MDA, a lipid peroxidation product, in the liver of high-fat-fed laying hens increased. More importantly, the antioxidant capacity of the liver also gradually decreased, which was manifested as a decrease in the activity of the antioxidant enzyme SOD. It was reported that phosphatidylcholine had good antioxidant activity and could be used as an antioxidant. Experiments showed that adding soybean lecithin to super palm oil could reduce the rate of oil oxidation (Pan et al., 2016). The current study revealed that long-term use of lecithin reduced the content of MDA (product of lipid peroxidation in the liver) resulting in an increase in the content of SOD, T-AOC, and GSH-Px in the liver, ultimately improving the antioxidant capacity of the liver of laying hens. Siyal et al. (2017) also mentioned that the addition of 0.05 and 0.1% soybean lecithin increased the activity of T-AOC, T-SOD, and GSH-Px in serum, and decreased the content of MDA in serum, which was consistent with the results in the current study. This might be because lecithin supplementation could repair cell membranes damaged by oxidative stress, increased the unsaturation of cell membrane fatty acids, and improved the metabolic, self-healing, and regeneration capabilities of cells.

## Conclusion

In conclusion, feeding high-fat diets adversely affects laying hens, while lecithin supplementation promotes fat absorption. When used for a long time, it can reduce the blood lipid and liver fat of laying hens. Meanwhile, it can improve the antioxidant capacity of the liver of laying hens. Therefore, adding lecithin has a positive effect on liver health.

## Data Availability

The raw data supporting the conclusions of this article will be made available by the authors, without undue reservation.
